# Multiscale Numerical Study of Enhanced Ductility Ratios and Capacity in Carbon Fiber-Reinforced Polymer Concrete Beams for Safety Design

**DOI:** 10.3390/polym17020234

**Published:** 2025-01-17

**Authors:** Moab Maidi, Gili Lifshitz Sherzer, Erez Gal

**Affiliations:** 1 Department of Civil and Environmental Engineering, Ben-Gurion University of the Negev, Beer Sheva 8410501, Israel; erezgal@bgu.ac.il; 2 Department of Civil Engineering, Braude College of Engineering, Karmiel 2161002, Israel; 3 Department of Civil Engineering, Ariel University, Ariel 4070000, Israel; gilil@ariel.ac.il

**Keywords:** ductility ratio enhancement, seismic performance, CFRP, high corrosion resistance, energy dissipation, performance, limited displacement, structural capacity, ductility index

## Abstract

Rigid reinforced concrete (RC) frames are generally adopted as stiff elements to make the building structures resistant to seismic forces. However, a method has yet to be fully sought to provide earthquake resistance through optimizing beam and column performance in a rigid frame. Due to its high corrosion resistance, the integration of CFRP offers an opportunity to reduce frequent repairs and increase durability. This paper presents the structural response of CFRP beams integrated into rigid frames when subjected to seismic events. Without any design provision for CFRP systems in extreme events, multiscale simulations and parametric analyses were performed to optimize the residual state and global performance. Macroparameters, represented by the ductility ratio and microfactors, have been analyzed using a customized version of the modified compression field theory (MCFT). The main parameters considered were reinforcement under tension and compression, strength of concrete, height-to-width ratio, section cover, and confinement level, all of which are important to understand their influence on seismic performance. The parametric analysis results highlight the increased ductility and higher load-carrying capacity of the CFRP-reinforced tested component compared to the RC component. These results shed light on the possibility of designing CFRP-reinforced concrete components that could improve ductile frames with increased energy dissipation and be suitable for applications in non-corrosive seismic-resistant buildings. This also shows reduced brittleness and enhancement in the failure mode. Numerical simulations and experimental results showed a strong correlation with a deviation of about 8.3%, underlining the reliability of the proposed approach for designing seismic-resistant CFRP-reinforced structures.

## 1. Introduction

Seismic events pose significant risks to buildings and their occupants. Conventional construction, which often relies heavily on steel reinforcement, consumes substantial natural resources and has a notable environmental impact [1,2,3,4,5,6,7,8,9,10]. In contrast, using CFRP for reinforcement presents a more sustainable alternative, reducing resource use and environmental footprint. Studies comparing these materials highlight CFRP’s advantages in terms of both efficiency and ecological impact [11]. The search for alternatives to steel bars in reinforced concrete is increasingly pertinent, yet the construction sector will unlikely abandon reinforced concrete technology anytime soon. Reinforced concrete remains a cornerstone of construction due to its numerous advantages. This material’s durability, cost-effectiveness, and versatility make it indispensable for various structural applications, from residential buildings to large infrastructures. Reinforced concrete’s ability to be molded into different shapes and its excellent fire resistance further solidify its status as a preferred construction material. However, innovations such as Carbon Fiber Reinforced Polymer (CFRP) and other advanced materials are being explored to address some of the limitations of steel reinforcement, particularly its susceptibility to corrosion, which can compromise the longevity and safety of concrete structures. Durability issues can be avoided by using fiber reinforced polymer (FRP) instead of steel. Fibers contribute to the tensile capacity of CFRP, as demonstrated in the studies by Maidi et al. [12].

Reducing the amount of CFRP in the compressive fiber can enhance the ductility of the component, while reducing the section depth may increase the curvature in the ultimate state of the section. Over-reinforcing the tensional fiber to support the compressive area can further improve the ductility of the section. The transition from deeper sections to flatter ones enhances ductility by increasing the component’s capacity for plastic deformation. However, increasing the percentage of tensioned reinforcement beyond 2% may create a yield strain in the compressed area, which can adversely affect the overall ductility of the section. Their work showed that higher tolerance and curvature can be achieved by decreasing the section depth and increasing its width, thereby creating an alternate compression area. These adjustments, appropriate reinforcement ratios, and concrete strength must be carefully considered when designing sections requiring high ductility to ensure optimal performance.

Providing shear and compressive capacities to overcome the limitations of CFRP material. Moreover, Maidi et al. [13] pointed out that corrosion-induced degradation in concrete and reinforced concrete (RC) structures often starts within the first few decades of their lifespan, and this remains a significant challenge to seismic resistance. Existing research tools can evaluate performance, but they usually fail to predict changes in seismic resistance resulting from alterations in the core properties of RC structures. To this gap, the research addresses the substitution of steel reinforcement with CFRP [14]. CFRP is a lightweight, high-strength material that enhances load-carrying capacity, stiffness, and ductility to create effective CFRP-reinforced concrete (CRC). However, under high stress or strain, CFRP can experience brittle failure, potentially compromising the structural integrity of the reinforced concrete member.

According to the model presented in Section 3, ductility is influenced by the curvature and failure mechanism, which ensures considerable strain in the compressed fiber. The increase in ductility is achieved by effective confinement and the use of relatively low concrete strengths. Therefore, improvements are necessary to enhance beam ductility and compare performance with traditional reinforced concrete (RC). Although CFRP does not corrode in the same manner as steel, prolonged water exposure, high temperatures, and high long-term stress can cause durability issues [15,16,17]. An alkali concrete environment can also attenuate the durability of some types of fiber, but CFRP is usually made of alkali-resistant materials. When compared to steel, CFRP has several disadvantages in structural applications, including lack of ductility and complicated bending of bars [18]. Although bending is possible during production, the strength of bent bars is lower and construction options are limited. CFRP bars also have poor performance when loaded in compression [19], due to stability issues.

In seismically active areas, material ductility is a property of greater importance than strength, for instance, seismic events [12,13,20,21,22,23]. Although ductility enables the design of safer structures, a nonlinear analysis complicates calculations, and design values [22]. On a structural level, capacity design is very important because it aims to avoid non-ductile types of failure such as shear. This is implemented in the current Croatian (and European) design code HRN EN 1998-1 [23,24], as well as in most other codes across the globe [25,26,27,28]. On an elemental level, detailing provisions can significantly influence element behavior. For example, the amount of compressive and confinement reinforcement can improve the behavior of elements [21], while increasing the tensile reinforcement can decrease their ductility. Elements that fail by crushing concrete before the steel reinforcement yields have a very low ductility, as in the case of over-reinforced beams.

One significant gap in the existing literature is the absence of an analysis that considers all relevant parameters collectively and assesses the extent of their impact on structural behavior. Such comprehensive studies are crucial for developing more accurate predictive models and enhancing construction materials’ effectiveness in real-world applications.

Research on the ductility of elements with CFRP rebars is limited. Most works have focused on the use of CFRP for structural repair and strengthening [29], rather than in new structures. In addition, several methods used to design/construct new elements are analyzed, as they relate well to CFRP-reinforced elements and provide crucial insight into the behavior of concrete elements [30]. This paper discusses the ductility of new structures, with emphasis on new beam structures, by examining parameters on ductility and shear ductility. The need to explore alternatives to steel as the primary reinforcement, such as various fibers, was highlighted in a study by Lu et al. (2022) [31]. Furthermore, as discussed in [12], recommendations for optimizing fiber performance were provided, focusing on reinforcement ratios, cross-sectional dimensions, concrete strengths, and concrete cover. These recommendations aim to inform and enhance design codes. When properly designed, combining reinforced concrete with CFRP offers a promising solution to bridging these gaps in structural performance and durability.

## 2. Methodology

This research focuses on analyzing the energy dissipation of flexural components under bending, investigating various macro, meso, and micro parameters through Push-Over Analysis (POA). The methodology employed is based on the approach presented in the study by Maidi and Shufrin [32]. The simulations are conducted using the Response-2000 program (v1.0), which incorporates the Modified Compression Field Theory (MCFT) model developed by Bentz in 2000 [33].

**Figure 1:**
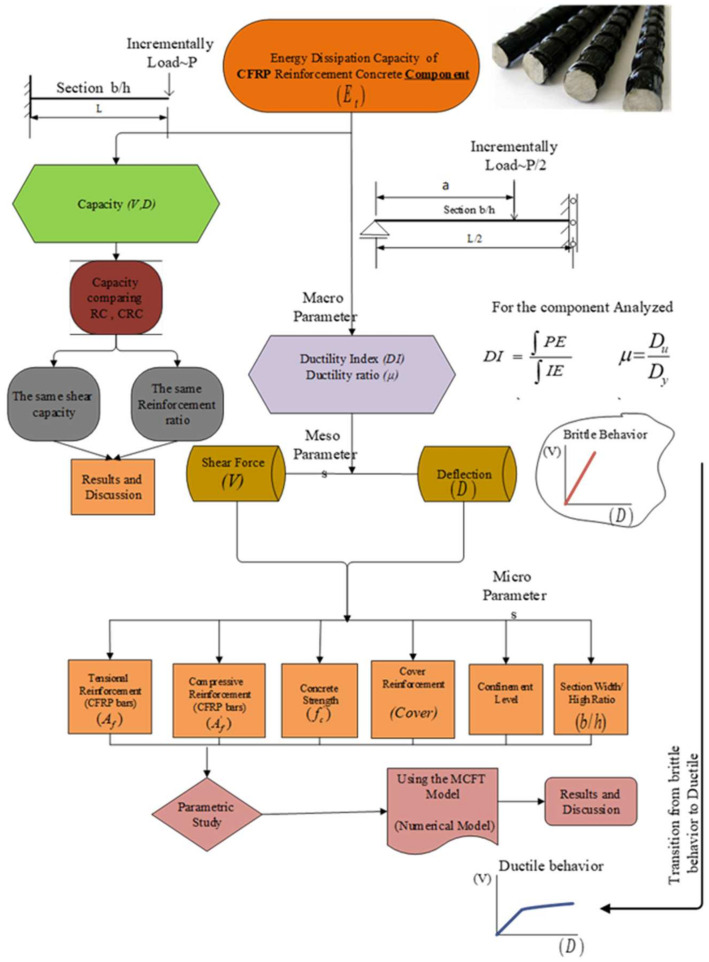
Methodology framework.

The macro parameters are characterized by ductility ratio (μ), or ductility index (*DI*) predict the residual state and are defined by two meso parameters: shear force (*V*) and displacement (*D*). The study also examines how micro parameters, such as tensile CFRP reinforcement (*Af*) and compressive CFRP reinforcement (*A′f*), depth (*d*), concrete strength (*f′c*), and its cover thickness (*df*), influence the macro parameters (*V*) and (*D*). A parametric study is conducted to analyze the behavior of the CFRP-reinforcement section, considering the interaction between macro, meso, and micro parameters. The study ultimately demonstrates improvements in flexural performance and energy dissipation, highlighting potential enhancements in seismic performance. The methodology framework is illustrated in Figure 1.

## 3. Multiscale Study

### 3.1. Macroscale Study (MASS)

**Figure 2:**
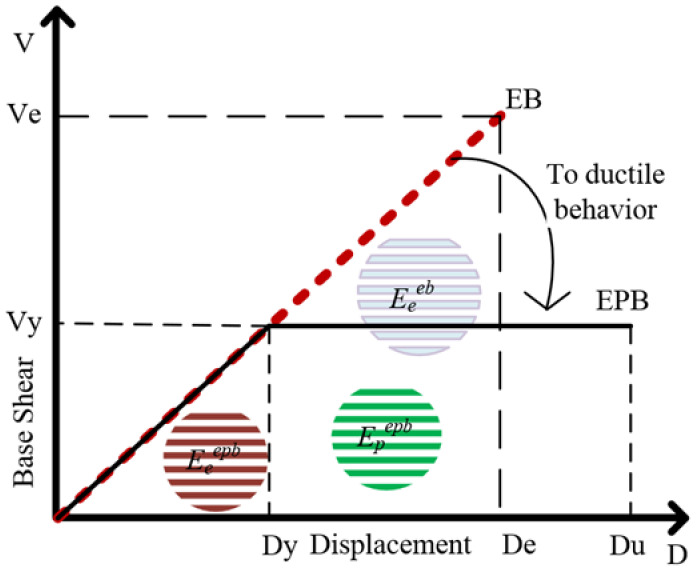
From Transition from brittle to ductile force–displacement behaviors.

The ductility ratio, which is considered a macroparameter, reflects the capacity of the tested bending component to dissipate maximum plastic energy, and is calculated as the ratio of elastic to plastic dissipation energies. The amount of dissipated energy is expressed using Equation (1):

(1) $$E_{t}=\int_{0}^{D_{u}}EPB\left(D\right)d\left(D\right)=E_{e}^{epb}+E_{p}^{epb}$$

where (EPB) represents the elastic-plastic behavior, (EB) represents the elastic behavior and *E_e_^eb^* is the total elastic energy in the elastic behavior. The following characteristics are presented in Figure 2, *V_e_*, De are the base shear and top displacement, respectively, in the elastic state. *D_y_*, *D_u_* correspond to the yield and ultimate state displacements, respectively, with *V_y_* denoting the shear force capacity in the ductile behavior. $E_{e}^{epb}$, $E_{p}^{epb}$ represent the elastic and plastic energies, respectively, in the ductile behavior.

The macroparameter, characterized as the ductility ratio *µ*, can then be expressed by Equation (2) [34]:

(2) $$\mu =\frac{D_{u}}{D_{y}}$$

### 3.2. Mesoscale Study (MESS)

The correlation between the yield state strain and the displacement *D_y_* in bending can be determined with Equations (3) and (4) [21]:

(3) $$\varphi_{y}=\frac{\varepsilon_{cy}+\varepsilon_{fy}}{d_{y}=d_{f}}$$

(4) $$D_{y}=\int_{0}^{L}x\cdot \varphi_{y}dx$$

where ε_cy_ denotes the strain ratio in concrete at yield state, ε_fy_ denotes the strain corresponding to the yield strength of CFRP reinforcement, defined as *f_fy_/E_f_*, and d denotes the distance from the extreme compression fiber to the centroid of tension reinforcement. Similarly, the ultimate state can be obtained by Equations (5)–(7):

(5) $$\varphi_{u}=\frac{\varepsilon_{cu}+\varepsilon_{fu}}{d_{u}}$$

(6) $$d_{u}=d_{f}-c$$

(7) $$D_{u}=\int_{0}^{L}x\cdot \varphi_{u}dx$$

where ε_cu_ denotes the strain in concrete at the ultimate state, and ε_fu_ denotes the strain corresponding to the ultimate strength of CFRP reinforcement, defined as f_fu_/E_f_.

Note: For the CFRP material, the strain value and the strengths at both the yield and ultimate states are equal, as illustrated in Section 4. 

### 3.3. Microscale Study (MISS)

This section describes the method used to analyze the impact of six microparameters on the performance of a doubly reinforced section (DRS) and a single reinforced section (SRS) under a theoretical moment-displacement representation framework. The DRS featured reinforcement bars at both the top and bottom of the beam, while the SRS had a single reinforcement section at the bottom. The analysis incorporated the displacement approach proposed by Kišiček et al. [22], the balanced stress–strain diagrams by Kiši, Sori, and Gali, the effects of confinement methods on the ductility of over-reinforced RC sections, as reported by Ahmed et al. [35] and the Concrete Compression Failure Principle (CCFP) outlined by Paulay and Park. Furthermore, the tensile failure limitation proposed by Bazan and Fernandez-Davila [34] was incorporated to evaluate failure in the compressive zone and facilitate the cross-section transition from brittle to ductile behavior. Additionally, the residual state of the CFRP was assessed through a four-point bending test, focusing on the influence of six microparameters, as outlined in Section 3.3.1, Section 3.3.2, Section 3.3.3, Section 3.3.4, Section 3.3.5 and Section 3.3.6 below.

#### 3.3.1. Tensional CFRP Reinforcement Effect

The failure mode in CFRP-reinforced concrete structures is significantly influenced by the amount of reinforcement in the tensioned area. Increasing the amount of CFRP reinforcement in this zone can lead to failure at the compressed fiber, which is generally a more desirable failure than brittle failure in the tension zone. Increasing the reinforcement in the compressed fiber can enhance the structure’s flexural and bending capacity. However, if the tensile reinforcement ratio exceeds the balanced failure ratio, further increases in tensile reinforcement will not yield additional benefits. The balanced reinforcement ratio was determined by varying the CFRP reinforcement areas.

#### 3.3.2. Compressive CFRP Reinforcement Effect

The ultimate displacement and moment were analyzed to understand the effect of double reinforced in both tension and compression sides.

#### 3.3.3. Effect of Concrete Strength

The ductility of a section is influenced by the strain level in the compressive stressed area, which is directly affected by the characteristic strength of the concrete. Consequently, this chapter examines the impact of concrete strength on the bending behavior of the section and the strain levels under different stress conditions. High concrete strength is expected to delay the onset of maximum strain in the concrete, which may negatively impact the overall ductility. This preliminary conclusion is supported by a work that showed that different types of concrete require different stress levels to achieve the same strain, as shown in Table 1 [21].

#### 3.3.4. Effect of Concrete Cover on Concrete Cracking

Concrete covers protect steel reinforcement from corrosion and fire resistance, and significantly influence the cracking behavior of concrete under various loads. Numerical analyses were conducted to investigate the impact of the concrete cover on cracking behavior. Specimens were subjected to four-point bending tests, and the relationship between crack width and spacing was analyzed. The Response-2000 numerical model used in this study is based on equations from both the American Concrete Institute [36,37] and Eurocode 2 [38], which establish a correlation between the spacing and width of concrete cracks, as shown in Equation (8):

(8) $$w=\varepsilon_{cm}s_{m}$$

where ε_cm_ denotes the average strain due to stress at a certain level, and sm denotes the crack spacing, which is calculated by Equation (6):

(9) $$s_{m}=3c_{max}$$

where $c_{max}$ denotes the distance from the furthest tension fiber to the nearest reinforcement, which is directly related to the cover thickness.

**Figure 3:**
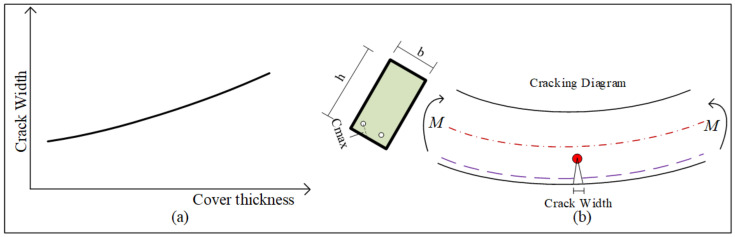
Typical relationships between the concrete cover thickness and crack width. (**a**) Schematic of crack width versus cover thickness curve. (**b**) Schematic diagram of crack opening in a beam.

Instead of increasing the distance c_max_, a multiplying factor *γ* can be applied to cmax, which is then incorporated into Equation (5) as follows: *w* = *ε_cm_γs_m_*. Then, the factor strain is multiplied by the factor γ, plugged into Equation (2), to enhance ductility. Therefore, increasing the cover thickness not only leads to a greater crack width but also improves ductility, as illustrated in Figure 3.

#### 3.3.5. Effect of Confinement Concrete Level

The strength and ductility of concrete are significantly influenced by the level of confinement provided by the vertical reinforcement within the beam’s structure. The pivotal roles played by the stiffness and behavior of the confining reinforcement, ranging from elastic to elastoplastic, are crucial in determining the overall performance of concrete [39]. This research incorporates a concrete confinement model to enhance the shear capacity and ductility ratios.

**Figure 4:**
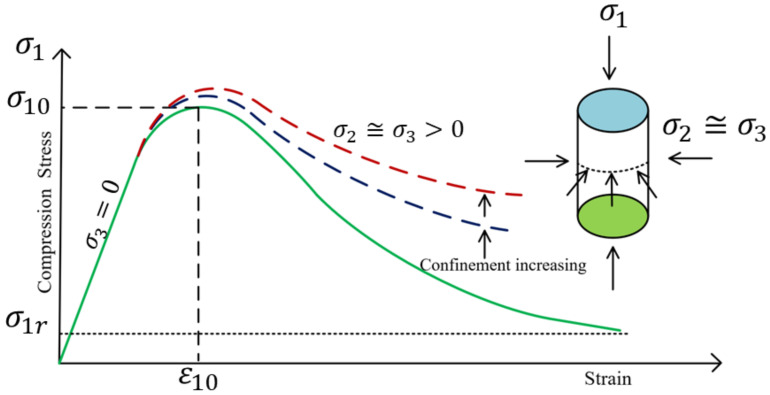
Constitutive model of confined concrete under uniaxial compression.

In the analysis framework, the axial and lateral directions are referred to as directions 1 and 3, respectively, assuming that stresses and strains are equivalent in both lateral directions (σ_2_ ≈ σ_3_). Compressive stresses and strains are considered positive, while volume expansion is negative. When confined by constant lateral pressure, the axial stress–strain response of concrete is characterized at three distinct points on the stress–strain curve, as outlined in the referenced study [40]. This methodological approach is visually represented in Figure 4, clearly depicting the model’s application to enhance our understanding of confined concrete behavior under various loading conditions.

The ultimate strength of the concrete, denoted as (σ_10_), is directly influenced by the lateral pressure applied, while the residual capacity, (σ_1r_), is determined by the internal friction remaining after initial stress effects. The analysis employs the Leon–Pramono [40], criterion as defined in Equation (10) to precisely characterize these critical points on the stress–strain curve:

(10) 1−kσ3fc′2+σ1−σ3fc′2+k2mσ3fc′−k2c=0

where k denotes the hardening parameter, defined as 0.1 at the elastic limit. The confinement modulus, m, as described in Equation (11), is calculated as follows:

(11) $$m=\frac{f_{c}^{\prime 2}\cdot f_{r}^{2}}{f_{c}^{\prime }\cdot f_{r}}$$

This equation simplifies the understanding of confinement effects on the compressive strength of concrete, where *ϕ* denoted as the confinement ratio, is calculated as follows:

(12) ϕ=σ3fc′

**Figure 5:**
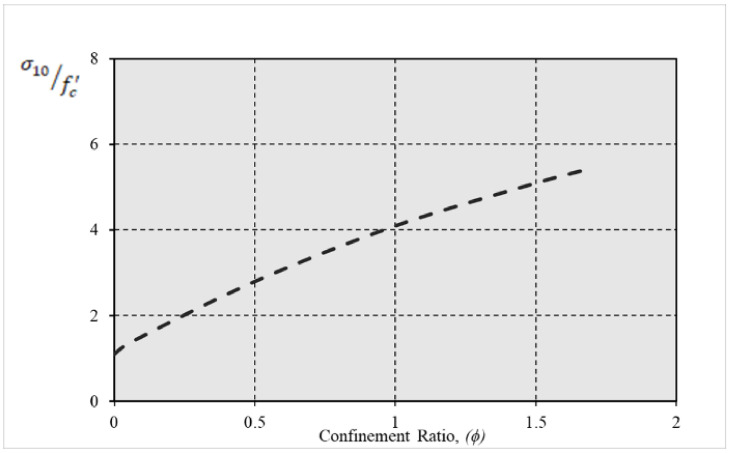
The increase ratio (i) in the strength vs. confinement ratios.

Finally, the increase in strength due to confinement, denoted as i, is calculated using Equation (13) and is illustrated in Figure 5:

(13) i=σ10fc′

#### 3.3.6. Effect of Section Dimensions Ratio

In this section, we demonstrate how the sensitivity of reinforcement amounts, concrete strength, and b/h ratios to determine the optimal dimension ratio with specific reinforcement ratios. The primary goal is to identify the optimum ratio between the height and width of the tested section, thereby establishing the ideal b/h ratio.

### 3.4. Modeling of Moment Displacement Curve

**Figure 6:**
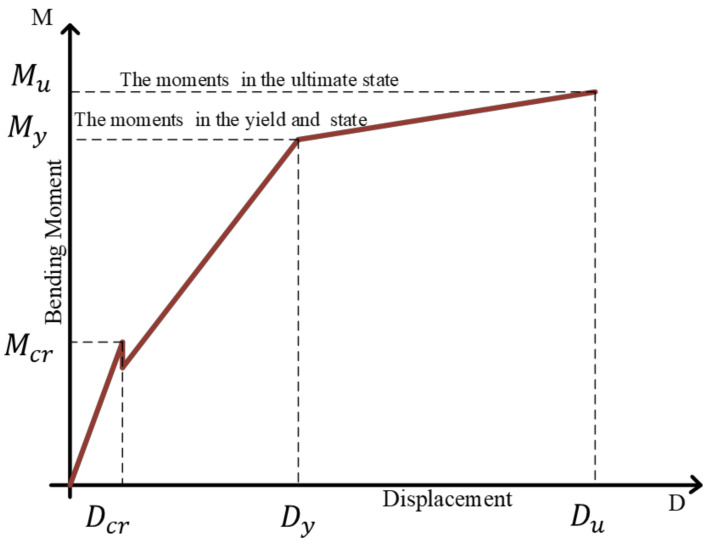
Schematic model of a moment displacement curve.

The effect of the ductility behavior is displayed in Figure 5, the moment-displacement curve can be schematically divided into three straight segments, which influence the ductility behavior. The key points on the moment-displacement curve are (*Dcr*, *Mcr*), (*Dy*, *My*), and (*Du*, *Mu*). The symbols used in deriving the moment-displacement model are illustrated in Figure 6.

**Figure 7:**
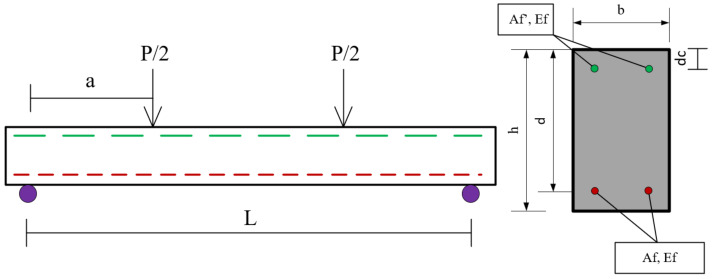
Beam dimensions and cross-section for four-point bending analyses.

The symbols used in deriving the moment-displacement model are illustrated in Figure 7.

The three main stages to obtain a capacity curve with the three classic control points are presented below.

Stage I: Elastic Stage (0 to Cracking Moment, *M_cr_*), This stage begins with no initial displacement and zero moment, and progresses to the cracking moment (*M_cr_*). The mid-span displacement increases from 0 to (*D_cr_*), corresponding to the cracking displacement. At this stage, the displacement can be computed using a linear elastic relationship up to *M_cr_*. Often this analysis is based on beam theory or specific structural behavior Equations (14)–(16). The general presentation is as follows:

(14) $$M_{cr}=\frac{I_{g}\cdot f_{r}}{y}$$

when the section is semitic at y axial:

(15) $$M_{cr}=\frac{2I_{g}\cdot f_{r}}{h}$$

(16) $$D_{cr}=\frac{M_{cr}}{24\cdot E_{c}\cdot I_{g}}\langle 3\cdot L^{2}-4\cdot a^{2}\rangle$$

where *I_g_* denotes the moment of inertia of the uncracked section, calculated using *I_g_* = *b × h* 3/12, where b denotes the width and h the height of the section. The flexural tensile strength of concrete, fr can be determined as *fr* = 0.7($f_{c}^{\prime })$ (MPa) for normal-weight concrete, where *f*′*c* is the concrete’s compressive strength and y is the distance between the natural axis and the extreme of the tensional zone.

Stage II: The maximum moment increases from the cracking moment *M_cr_* to the yielding moment, corresponding to concrete yielding *M_y_*, the mid-span displacement increases from *D_cr_* to *D_y_*.

**Figure 8:**
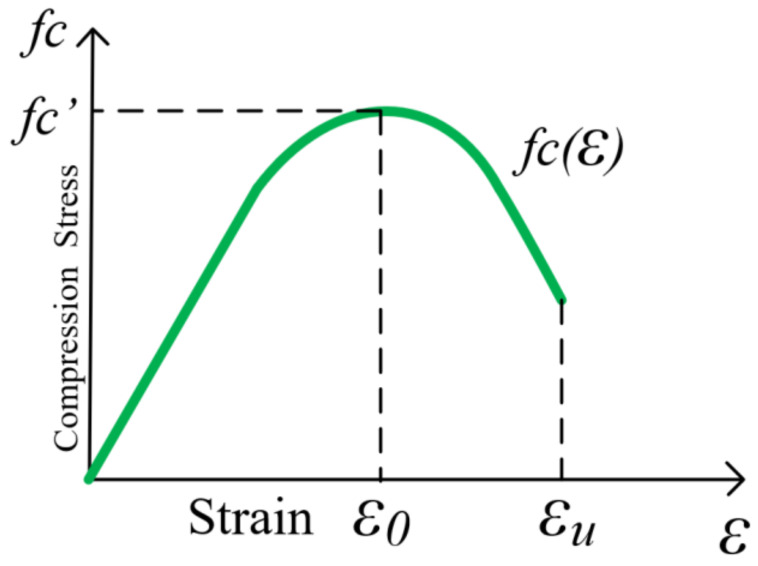
Constitutive model of concrete subjected to uniaxial compression for a non-confined condition.

**Figure 9:**
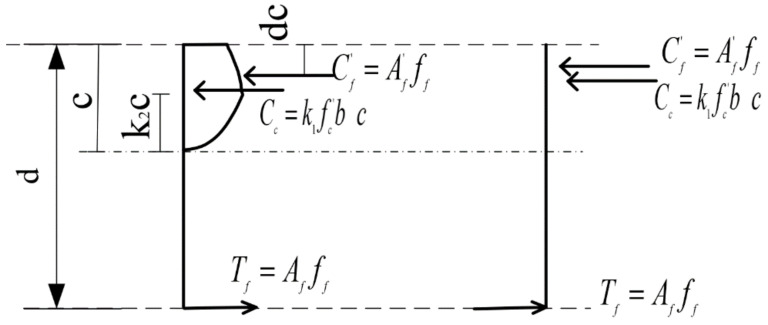
Stresses block and force distribution in the beam cross-section.

A generally accepted constitutive model used for concrete in compression Figure 6 assumes that the strain distribution along the depth of the beam section remains linear. Concrete contribution to tension is negligible in the derivation, which simplifies the analysis. According to Figure 7, the total compressive force from the concrete can be given by Equations (17) and (18).

(17) $$C_{c}=\int_{0}^{\varepsilon_{c}}f_{c}\left(\varepsilon \right)\cdot b\cdot c/\varepsilon_{c}d\varepsilon$$

where ε_c_ denotes the concrete strain at the extreme compression fiber and c denotes the height of the concrete compression block. The solution to Equation (17) is presented in Equation (18) as a simplified expression for the total compressive force *C_c_*.

(18) $$C_{c}=k_{1}\cdot f_{c}^{\prime }\cdot b\cdot c$$

where *k*_1_ is a coefficient that accounts for the shape of the stress distribution in the compression zone and c is the depth of the compression block as presented in Equation (19) and Figure 8 and Figure 9.

(19) $$f_{c}(\varepsilon )=f_{c}\prime \left[2\cdot \left(\frac{\varepsilon }{\varepsilon_{0}}\right)-{\left(\frac{\varepsilon }{\varepsilon_{0}}\right)}^{2}\right]$$

The total bending moment, *M_c_*, provided by the concrete with respect to the neutral axis is expressed by Equations (20)–(22).

(20) $$M_{c}=\frac{\int_{0}^{\varepsilon_{c}}f_{c}\left(\varepsilon \right)\cdot {b\cdot c}^{2}\cdot \varepsilon }{\varepsilon_{c}^{2}d\varepsilon }=k_{1}\cdot k_{2}\cdot f_{c}^{\prime }\cdot b\cdot c^{2}$$

(21) $$k_{1}=\frac{1}{f_{c}^{\prime }\cdot \varepsilon_{c}}\int_{0}^{\varepsilon_{c}}f_{c}\left(\varepsilon \right)d\varepsilon =\frac{\varepsilon_{c}}{\varepsilon_{0}}\cdot \left(1-\frac{1}{3}\cdot \frac{\varepsilon_{c}}{\varepsilon_{0}}\right)$$

(22) $$k_{2}=\frac{1}{\varepsilon_{c}}\frac{\int_{0}^{\varepsilon_{c}}f_{c}\left(\varepsilon \right)\cdot \varepsilon d\varepsilon }{\int_{0}^{\varepsilon_{c}}f_{c}\left(\varepsilon \right)d\varepsilon }=\frac{2}{3}\left(\frac{1-\frac{3}{8}\cdot \frac{\varepsilon_{c}}{\varepsilon_{0}}}{1-\frac{1}{3}\cdot \frac{\varepsilon_{c}}{\varepsilon_{0}}}\right)$$

Typically, $\varepsilon_{0}$ = 0.002, which allows *k*_1_ and *k*_2_ to be presented as follows:

(23) $$k_{1}=500\cdot \varepsilon_{c}\langle 1-166.7\cdot \varepsilon_{c}\rangle$$

(24) $$k_{2}=\frac{\langle 2-375\cdot \varepsilon_{c}\rangle }{\langle 3-500\cdot \varepsilon_{c}\rangle }$$

Thus, the yielding moment *M_y_* is given by Equation (25).

(25) $$M_{y}=k_{1}\cdot k_{2}\cdot f_{c}^{\prime }\cdot b\cdot c^{2}+A_{f}^{\prime }\cdot E_{f}\cdot \varepsilon_{f}^{\prime }\left(c-d_{c}\right)+A_{f}\cdot f_{f}\cdot \left(d-c\right)$$

where *A′_f_*, *E_f_*, *ε′_f_*, *d_c_*, *A_f_*, and ff represent the CFRP area at the top fiber, modulus of elasticity, top fiber strain, the active depth, CFRP area at the bottom fiber, and forces associated with reinforcement.

The mid-span displacement at yielding, Dy, can then be calculated by the following:

(26) $$D_{y}=\frac{M_{y}}{24\cdot E_{c}\cdot I_{eff,y}}\langle 3\cdot L^{2}-4\cdot a^{2}\rangle$$

or in terms of curvature φ_y_ by the following:

(27) $$D_{y}=\frac{\varphi_{y}}{24}\langle 3\cdot L^{2}-4\cdot a^{2}\rangle$$

The effective moment of inertia at yielding (*I_eff_*_,*y*_) is given by the following:

(28) $$I_{eff,y}={\left(\frac{M_{cr}}{M_{y}}\right)}^{3}\cdot I_{unc}+\left(1-{\left(\frac{M_{cr}}{M_{y}}\right)}^{3}\right)\cdot I_{cr}$$

where *I_unc_* denotes the uncracked second moment of area, calculated by the following:

(29) $$I_{unc}=\left(\frac{1}{12}*b*h^{3}\right)+b\cdot h{\left(y^{\prime }-\frac{h}{2}\right)}^{2}+\left(\frac{E_{f}}{E_{c}}-1\right)\cdot A_{f}\cdot {\left(d-y^{\prime }\right)}^{2}$$

The moment of inertia *I_cr_* for the cracked section can be derived using the transformed area method, as follows:

(30) $$I_{cr}=\frac{1}{3}b\cdot c^{3}+\frac{E_{f}}{E_{c}}\cdot A_{f}{\langle d-c\rangle }^{2}$$

where $E_{c}=4730\sqrt{f_{c}^{\prime }}$ (MPa), as specified by ACI 318-05 [36]. In calculating displacements, the stress–strain relationship for concrete in compression is assumed to be linearly elastic, so long as the concrete’s extreme fiber remains within the ascending branch of the curve shown in Figure 6.

Stage III: As the maximum moment increases from the yielding moment *M_y_* to the ultimate moment *M_u_*, the mid-span displacement increases from *D_y_* to *D_u_* calculated by:

(31) $$M_{u}=k_{1}\cdot k_{2}\cdot f_{c}^{\prime }\cdot b\cdot c^{2}+A_{f}\cdot E_{f}\cdot \varepsilon_{ff}\left(d-c\right)$$

where *k*_1_, *k*_2_, and c are determined by the same equations as in stage II, and *εff* is the CFRP strain at beam failure. If failure occurs due to CFRP rupture, *εff* is taken as the ultimate CFRP strain, *εfu*, so *εff* = *εfu.*

After CFRP failure, the displacement Du can be given by the following:

(32) $$D_{u}=\frac{M_{u}}{24\cdot E_{c}\cdot I_{eff,u}}\langle 3\cdot L^{2}-4{\cdot a}^{2}\rangle$$

where the effective moment of inertia at ultimate (*I_eff_*_,*u*_) is as follows:

(33) $$I_{eff,u}={\left(\frac{M_{cr}}{M_{u}}\right)}^{3}\cdot I_{unc}+\left(1-{\left(\frac{M_{cr}}{M_{u}}\right)}^{3}\right)\cdot I_{cr}$$

The following are used to calculate the ultimate displacement:

(34) $$\varphi_{u}=\frac{M_{u}}{E_{c}\cdot I_{eff,u}}$$

(35) $$D_{u}=\frac{\varphi_{u}}{24\cdot E_{c}}\langle 3\cdot L^{2}-4\cdot a^{2}\rangle$$

The moment can be converted to an equivalent shear force using the following:

(36) $$P=\frac{{2M}_{max}}{a}$$

This procedure enables the construction of a shear force-displacement curve for the selected beam, allowing for detailed examination of the beam’s behavior under the specified parameters.

## 4. Materials Characterization

This study incorporates a steel rebar force equivalent to that of the compressed section. The concrete properties and steel bars were selected based on the guidelines outlined in (CSA S806-12 2012) [27], while the properties of CFRP were based on those reported in studies by Suriyati et al. and Zhou et al. [41,42,43].

### 4.1. Concrete

Concrete properties significantly influence the behavior and performance of reinforced structures under various loading conditions. To ensure accurate modeling, different types of concrete were selected based on established studies. The mechanical properties of these concrete types are summarized in Table 1, as reported by Suriyati et al. and Zhou et al. [41,42,43].

The stress–strain relationship for different types of concrete is typically represented by a standard curve, as provided in ACI 318 (1995) [36].

### 4.2. Steel Bars

The properties of the steel reinforcement bars incorporated into the structural models were selected based on standardized specifications. The specific properties of the steel bars used in this study are detailed in Table 2.

### 4.3. CFRP Bars

**Figure 10:**
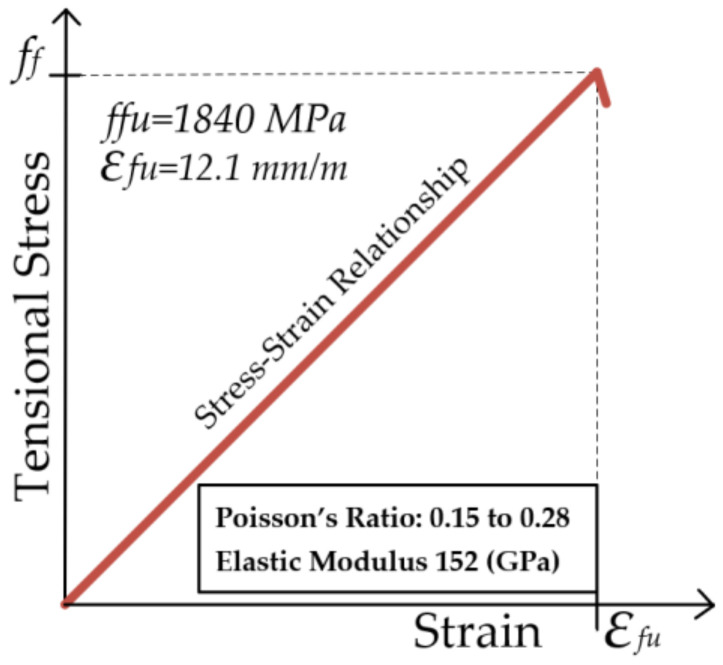
Mechanical properties of the CFRP reinforcement material.

CFRP is a high-strength, lightweight reinforcement material known for enhancing the load-carrying capacity, stiffness, and ductility of concrete structures. The mechanical properties of the CFRP used in this study are presented in Figure 10.

In addition to its high strength and lightweight properties, CFRP has a low density of approximately 1.60 × 10^−4^ N/mm^3^ and a thermal expansion coefficient of 40 × 10^−6^ °C. In this study, the diameter of the CFRP bars was selected to match that of the steel rebar. The brittle behavior of CFRP bars is illustrated in Figure 8. The properties of the CFRP used in this study are presented in Figure 10.

## 5. Results

### 5.1. Load-Bearing Capacity of CFRP-Reinforced Concrete Elements

**Figure 11:**
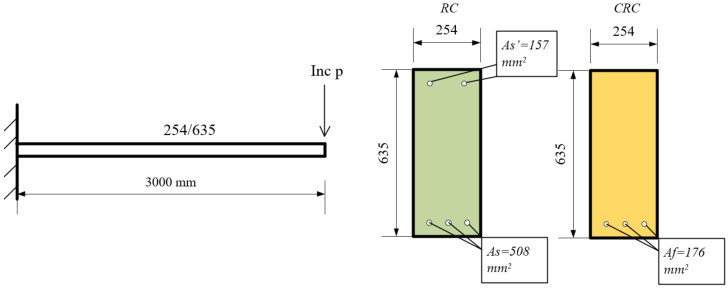
Schematic of RC and CRC Components Under Loading.

Two cantilever beam configurations (see Figure 11), both with the same cross-section and concrete strength, where one beam was reinforced with a steel bar and the other with CFRP, were compared.

First, a test was performed to determine the equivalent CFRP area value in the CRC component required to achieve the same capacity as the RC component. A second test compared the capacities of the two components with the same preset reinforcement values. In both scenarios, the ductility and effective stiffness, as defined by Equations (2) and (37), were calculated as follows:

(37) $$k_{eff}=\frac{V_{u}}{D_{u}}$$

**Figure 12:**
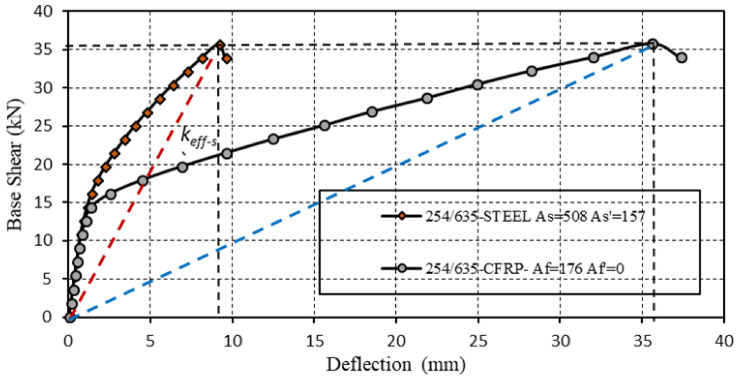
RC and CRC components equal capacity in the ultimate state, the blue and the red line are the effective stiffnesses.

For the first component, the cantilever span was 3000 mm, with a cross-sectional depth of 635 mm and a width of 254 mm. The concrete strength was *fc′* = 30 N/mm^2^, and the cover dimensions were *ds*, *ds′*, *df*, *df′* = 51 mm. The POA indicated that the reinforcement areas were suitable, with the top reinforcement areas measuring *As′* = 157 mm^2^ and *Af′* = 0 mm^2^, and the bottom reinforcement areas measuring *As* = 508 mm^2^, *Af* = 176 mm^2^. The reinforcement area was determined by comparing the capacities of the two numerical models. The POA calculated capacity under monotonic loading. While these values provided equal capacities for the two components, they resulted in different ductility levels, as illustrated in Figure 12.

The ductility of the CRC component was significantly higher than that of the RC component (15.33 and 8.77, respectively). The load-bearing capacities of the cross-sections were similar, with the steel-reinforced section achieving *Vus* = 35.634 kN and the CFRP-reinforced section achieving *Vuf* = 35.766 kN. However, the effective stiffness differed considerably; the steel-reinforced section had an effective stiffness of *keff-s* = 3.90 kN/m, while the CFRP-reinforced section had an effective stiffness of *keff-f* = 1.0065 kN/m. In conclusion, with the same capacity in both components, the concrete element with steel reinforcement exhibited greater effective stiffness but lower ductility. In contrast, the ductility parameter was relatively high with CFRP reinforcement. To achieve the same capacity as the RC component, the required amount of CFRP reinforcement in the CRC component was 176 mm^2^, compared to 665 mm^2^ steel reinforcement in the RC component. Namely, the amount of reinforcement with CFRP required constitutes 74% lower of the steel reinforcement area. Of note, when the carbon fibers were only introduced into the bottom area, the level of ductility in both situations was in favor of the CRC component, as presented in Figure 12. Additionally, it had better ductility than the same section with both bottom and top reinforcement. The ductility level in the RC component (8.77) was significantly lower than in the CRC component (15.33). The load-bearing capacities of the cross-sections were similar, with the steel-reinforced section achieving *Vu*,*s* = 35.634 kN and the CFRP-reinforced section achieving *Vu*,*f* = 35.766 kN. However, the effective stiffness differed considerably; the steel-reinforced section had an effective stiffness of *keff-s* = 3.90 kN, while the CFRP-reinforced section had an effective stiffness of *keff-f* = 1.0065 kN.

**Figure 13:**
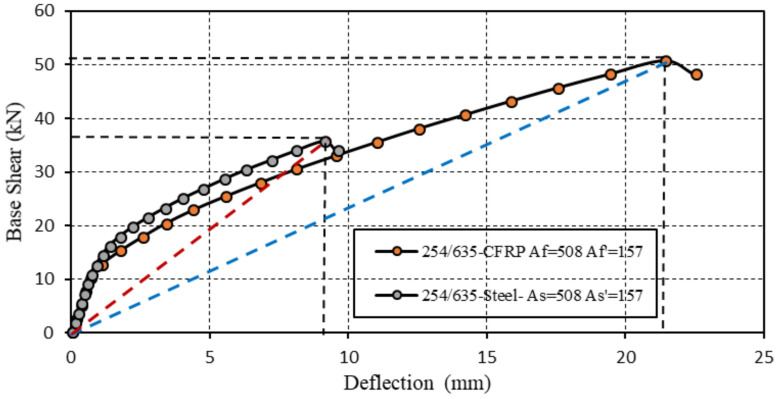
RC and CRC components with the same reinforcement ratios, the blue and the red line are the effective stiffnesses.

In the following situation, the cantilever span was also 3000 mm, with a cross-sectional depth of 635 mm, a width of 254 mm, and concrete strength of *fc′* = 30 N/mm^2^. The cover dimensions were ds, ds′, df, df′ = 51 mm, the top reinforcement areas were *As′*, *Af′* = 157 mm^2^, and the bottom reinforcement areas were *As*, *Af* = 508 mm^2^. POA demonstrated different capacities and ductility levels for the RC and CRC components, as shown in Figure 13.

Figure 13 shows the force-displacement curves for a beam with a 3000 mm span, where the orange curve represents the CRC cross-section, and the gray curve represents the RC section. The ductility level at the component was defined as the ratio between the displacement in the yielding state and the displacement in the failure state. The ductility level of the RC component was 8.77, compared to 15.87 for the CRC. Shear force at failure (ultimate state) was *Vus* = 33.853 kN for the RC component and *Vuf* = 50.663 kN for the CRC component. The effective stiffness, defined as the ratio between the shear force and horizontal displacement in the ultimate state, was *keff-s* = 3.53 kN/m for the steel-reinforced beam compared to *keff-f* = 2.365 kN/m for the same section reinforced with CFRP.

### 5.2. Sensitivity Analysis

**Figure 14:**
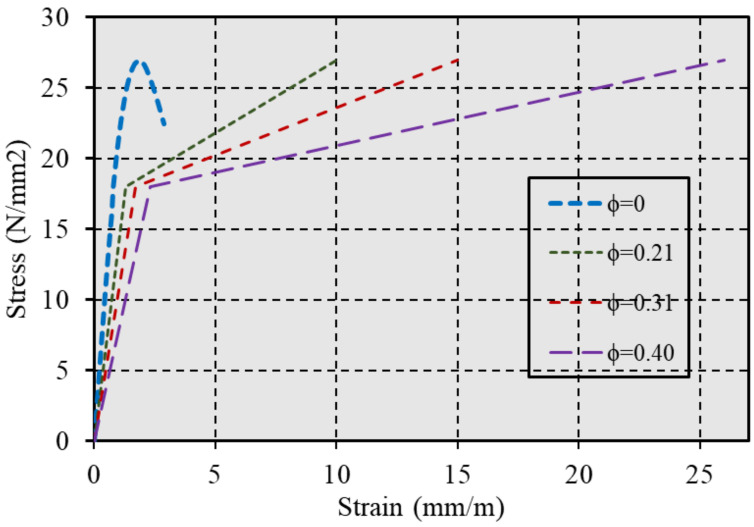
Stress–strain of variable confined concrete with a compressive strength of (*fc′* = 27).

Six microparameters were projected to significantly impact on the ductility of a beam section under bending: the amount of CFRP used in the tension and compression zones, the geometry of the beam cross-section, concrete cover, the concrete strength and confinement ratio as presented in Figure 14. A single beam section with a b/h ratio of 200/400 mm and with different cross-sectional geometries was used to evaluate the effects of the first three microparameters. To identify an optimal ductile section for shear loading, a series of beam sections, starting with a b/h ratio of 200/600 mm and progressing to a section with a b/h ratio of 600/200 mm, was examined.

#### 5.2.1. Single Reinforcement

**Figure 15:**
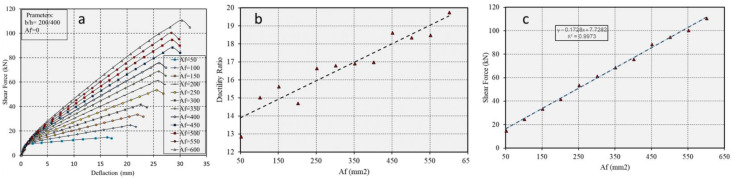
(**a**) Force-displacement curves for the beam, (**b**) ductility ratio, and (**c**) shear capacity as a function of different amounts of CFRP in the tension zone.

The effect of increasing amounts of CFRP in the tension zone on displacement was tested on a single beam with constant CFRP compressive reinforcement *Af′* = 0 and concrete compressive strength *fc′* = 30 N/mm^2^, but with varied bottom reinforcement. Figure 15a–c in the results section present the outcomes of this analysis.

#### 5.2.2. Double Reinforcement

**Figure 16:**
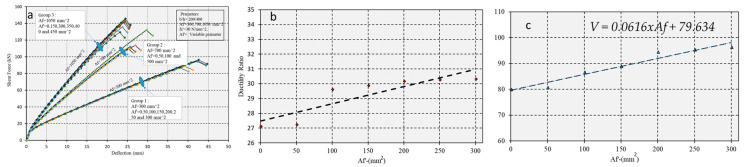
(**a**) Influence of varying amounts of CFRP on the force-displacement curve for a beam in the compressive zone. (**b**) Ductility ratio as a function of different amounts of tensioned CFRP. (**c**) Shear capacity variation in response to different amounts of compressive CFRP.

The effect of increasing amounts of CFRP in the compressive zone on bending behavior was measured in a beam with tensile reinforcement areas held constant at *Af* = 300,700 mm^2^ and *Af* = 1050 mm^2^, varied Af′ in the compressive zone, and a constant concrete strength of *fc′* = 30 N/mm^2^. The results of the analysis are shown in Figure 16a–c.

#### 5.2.3. The Role of Concrete Strength

**Figure 17:**
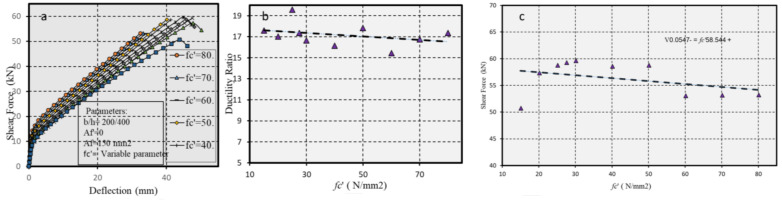
(**a**) Force-displacement curves with different of concrete strength, (**b**) ductility ratio, and (**c**) shear capacity for beams with varying concrete strengths.

The impact of concrete strength on the ductility ratio specifically in cases with no compressive reinforcement (*Af′* = 0) and with tensile reinforcement (*Af* = 150 mm^2^) was tested while varying the compressive strength *fc′*. Figure 17a–c presents the observations and corresponding data.

#### 5.2.4. Role of Cover Thickness in Enhancing the Durability of CFRP RC Structures

**Figure 18:**
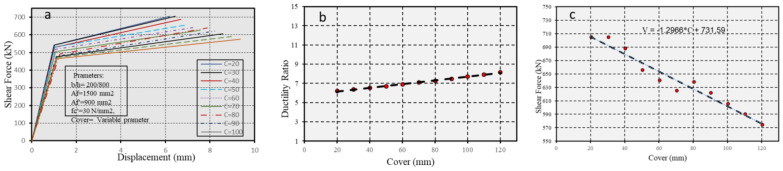
(**a**) Force-displacement curves with different of concrete cover, (**b**) ductility ratio, and (**c**) shear capacity as a function of different cover thickness.

To explore the relationship between the distance separating strain centers and deflection under pure flexure in the inspected section, the effect of cover thickness on a rectangular section with dimensions of 200 mm by 800 mm was characterized. The concrete strength was *fc′* = 30 MPa, with reinforcement at the top (*Af′* = 900 mm^2^) and bottom (*Af* = 1500 mm^2^). The primary variable under investigation was the cover thickness, as outlined in Equations (5) and (6). This parameter influences the moment-displacement relationship and the ductility ratio, as illustrated in Figure 18a–c.

#### 5.2.5. Effect of Confinement on Concrete Behavior

The effect of increasing the passive confinement ratio (ϕ) in the compressive zone of the bending component on ductile behavior was tested, with confinement parameters in the compressive zone applied at four levels, labeled from 1 to 4, as presented in Figure 14.

**Figure 19:**
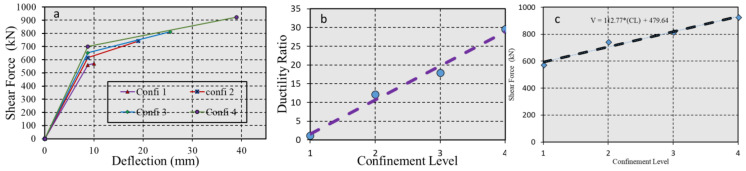
(**a**) Force-displacement curve, (**b**) ductility ratio, and (**c**) shear capacity as a function of confinement.

A rectangular section with dimensions of 200 mm by 500 mm was used, with Af = 800 mm^2^, Af′ = 0, and concrete compressive strength of fc′ = 27 N/mm^2^ held constant, while the stress–strain models for concrete were varied. The results of the analysis are presented in Figure 19a–c in the Results Section.

#### 5.2.6. Contribution of Section Dimensions Ratio

**Figure 20:**
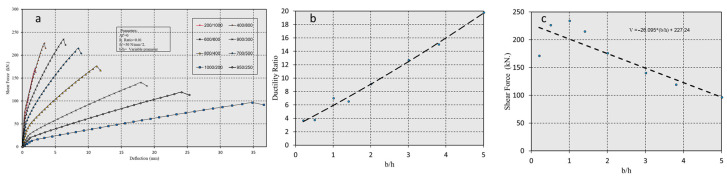
(**a**) Force-displacement curve, (**b**) ductility ratio, and (**c**) shear capacity for varying confinement ratios.

Considering Equations (1) and (2), it can be inferred that the depth of the cross-section being tested may impact both the curvature and ductility ratio. Therefore, the effect of the b/h ratio of the tested section was assessed while maintaining a constant reinforcement ratio of 0.10 mm² and a concrete compressive strength of fc′ = 30 MPa. Figure 20a–c showcases the results of this analysis.

### 5.3. Sensitivity Analysis Results

This section summarizes all results from Section 5, including shear force-displacement curves and ductility ratios, as functions of the tested parameters (see Figure 15, Figure 16, Figure 17, Figure 18, Figure 19 and Figure 20). Figure 15 presents the single reinforcement effective ductility.

Figure 16 presents the double reinforcement effective ductility.

Figure 17 presents the role of concrete strength effective ductility.

Figure 18 presents the role of cover thickness in enhancing the durability of CFRP and RC structures effective ductility.

Figure 19 presents the effect of confinement on concrete behavior effective ductility.

Figure 20 presents the section dimensions ratio effective ductility.

Figure 15, Figure 16, Figure 17, Figure 18, Figure 19 and Figure 20 present summarized simulation results representing various tests, illustrating shear force-deflection and ductility as functions of the tested parameters. Figure 15a mainly shows how increasing the amount of CFRP in the tensional fibers of a concrete member significantly enhances its ductility. Notably, while improving ductility, CFRP is susceptible to brittle failure under high levels of stress or strain, potentially compromising the structural integrity of the concrete element. To prevent such brittle failure, it is advisable to use more CFRP than typically required to achieve the concrete’s ultimate strength. This approach ensures that the CFRP reinforcement remains within its elastic and elastic-plastic ranges, preventing abrupt failures and enhancing the structure’s resilience. Adopting this over-reinforcement strategy ensures that failures if they occur, are more likely in the compressed fibers.

Additionally, the high stiffness of CFRP reduces displacement in the compressed area, further decreasing the likelihood of brittle failure. Figure 15b demonstrates that the amount of tensioned CFRP increases with increasing ductility of the concrete. Figure 15c shows a tendency for increased shear capacity with more tensioned CFRP. For the typical stress–strain behavior of CFRP, refer to Figure 10.

In Figure 16, the reduction in CFRP in the compressive fiber modestly enhances both ductility and shear capacity. Figure 17 highlights that higher concrete strengths increase yielding stress but predispose structures to more brittle failure modes than those with lower strengths. The least strong concrete showed the lowest yielding stress but was more ductile.

Figure 18 and Figure 19 reveal an interesting dynamic: as the cover thickness increases, the ductility ratio increases, but shear capacity decreases. Enhanced concrete confinement, as shown in Figure 19 and Figure 20, leads to higher ductility ratios and shear capacities. These enhancements suggest a critical balance between confinement and cover adjustments to optimize ductility and strength in seismic conditions.

Finally, Figure 20 underscores that an increase in the breadth-to-height (b/h) ratio not only improves ductility but also enhances the structure’s ability to absorb seismic energy, thereby boosting overall durability. The structural adjustments that enhance the b/h ratio lead to increased curvature at yield and ultimate states, significantly amplifying the ductility.

This comprehensive analysis is summarized in Table 3.

## 6. Validation

**Figure 21:**
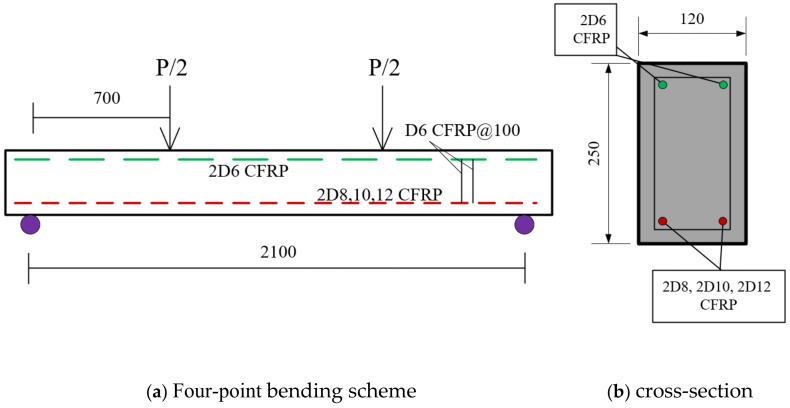
Section geometry and schemes: Illustrated are the dimensions of the CFRP-reinforced concrete sections, where D denotes the diameter of the CFRP bars. This specific section geometry was adapted from Wang et al. [44] study.

A specific concrete section reinforced with CFRP (Table 4), identified as (C82) and studied by Wang et al. [44], was chosen for validation of the numerical simulations performed with the Response-2000 software, developed by Bentz (2000) [33]. A critical aspect of the validation involved analysis of the load-deflection curve (LDC) associated with the section, as depicted in Figure 21. The systematic approach used for validation ensured that the simulations accurately mirror the behavior of CFRP-reinforced concrete sections under various loading conditions, thereby boosting the reliability and practical utility of the Response-2000 software for structural engineering analyses.

**Figure 22:**
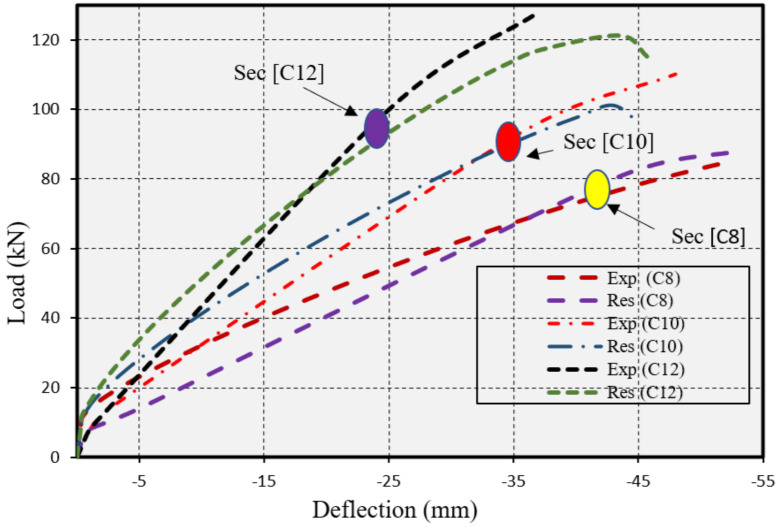
Load-deflection curves comparison using Response-2000 for the CFRP-reinforced section by Wang et al.

Figure 22 presents the Load-Deflection Curves (LDC) for CFRP-Reinforced Concrete Section: Illustrated is the comparison and validation of the performance under load of the concrete section studied by Wang et al. (2021) [44]. The red line denotes numerical results, and the magenta line represents experimental outcomes.

Figure 22 shows that the numerical analyses agreed well with the test results of the CFRP-strengthened segment. Such agreement of the numerical and experimental data verified the accuracy and applicability of Response-2000 in engineering practice, especially in design and analysis involving reinforced concrete members. Discrepancies may arise due to inaccuracies in the specimen’s size, support conditions, loading velocities, or other relevant factors.

## 7. Conclusions and Practical Recommendations

This research proposed a strategy to enhance structural durability against seismic forces by incorporating CFRP for concrete reinforcement. Through comprehensive multiscale simulations and evaluations, the study demonstrated that CFRP, despite its brittleness, can significantly improve the ductility of beam components in concrete structures. Key findings from this investigation can be summarized as follows:

- Increasing the amount of CFRP in the tensional zone in CRC components boosts both shear capacity and ductility ratios.
- Additional CFRP in the compressive zone, when matched with an equivalent amount in the tensional zone, does not significantly affect ductility or shear capacity.
- Lower concrete compressive strength can increase ductility and reduce brittleness and capacity.
- Increasing cover thickness enhances ductility ratios while reducing capacity.
- Decreasing the section depth can lead to increased curvature in the ultimate state of the section, and over-reinforcing the tensional fiber ensures that the compressive area improves the section’s ductility while decreasing its capacity.
- Enhancing the confinement ratio in concrete compression zones increases ductility and capacity while mitigating brittleness.
- The ductility of CRC cross-section is twice that of RC with identical concrete strength and reinforcement area.
- The shear capacity of CRC and RC structures is similar when the CFRP-to-steel ratio is 26.4%.

This study contributes to the ongoing effort to develop a user-friendly and accessible design methodology for practicing engineers. The recommendations are presented below as follow:

- Increase the amount of CFRP reinforcement in the tensional zone of CRC components to improve both shear capacity and ductility ratios.
- Avoid unnecessary CFRP in the compressive zone except when matched by equivalent amounts in the tensional zone, as this has minimal impact on ductility and capacity.
- Use lower concrete compressive strength to improve ductility and reduce brittleness, recognizing the trade-off with capacity.
- Consider using reduced section depth to increase curvature at the ultimate state, which improves ductility when over-reinforcing the tensional fiber.
- The compressive area should be adequately reinforced to balance ductility with capacity.
- Increase the thickness of the concrete cover provided, which increases the ductility ratios but most likely reduces the capacity.
- Increase confinement ratios in concrete compression zones to reduce brittleness and increase ductility and capacity.
- Design the CRC component with a CFRP-to-steel ratio of about 26.4% for a similar shear capacity to the RC structure.
- Make use of the higher ductility of the CRC sections, up to twice that of the identical concrete strength and area of reinforcement of RC sections.

## Figures and Tables

**Table 1:** Mechanical properties of tested concrete.

Concrete Type	Tension Strength (MPa)	Peak Strain (mm/m)	Cylinder Strength (MPa)	Modulus of Elasticity (MPa)
1	1.69 (2.83)	1.87	20.7	21,946.2
2	1.61	1.87	15	19,550.9
3	1.68	1.86	20	21,678.8
4	1.75	1.9	25	23,476.9
6	1.82	1.96	30	29,826.8
7	1.96	2.1	40	30,006.6
8	2.1	2.25	50	30,375.8
9	2.24	2.39	60	32,616.6
10	2.52	2.37	80	36,594.9

**Table 2:** Mechanical properties of the steel bars.

Properties	Ultimate Strain (m/mm)	Yield Strength (MPa)	Ultimate Strength (MPa)	Elastic Modulus (GPa)
Steel bars	7	276	414	200

**Table 3:** Four-point bending of parameters affecting ductility and their level of influence.

Variable Parameter	Ductility Ratio	Shear Capacity
Single reinforcement	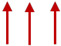	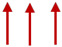
Double reinforcement	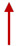	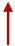
Concrete Strengthening	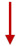	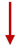
Cover thickness	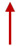	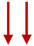
Confinement concrete ratio	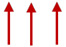	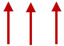
Section dimensions’ ratio	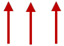	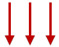

An arrow pointing up indicates improvement and a downward arrow indicates decline. The number of arrows indicates the intensity of the change.

**Table 4:** Properties of CFRP RC Sections.

Symbol	(C8)	(C10)	(C12)
Span *L* (mm)	2100	2100	2100
*a* (mm)	700	700	700
*b* (mm)	120	120	120
*h* (mm)	250	250	250
*Af* (bars)	2D8	2D10	2D10
*Af′* (bars)	2D6	2D6	2D6
*Ef* (MPa)	106.4	106.4	106.4
*Ffu* (MPa)	1628	1628	1628
*f′c* (GPa)	42.6	42.6	42.6
*Ec* (GPa)	31.6	31.6	31.6
*Cover* (mm)	25	25	25
ρ *f*	0.37%	0.58%	0.84%

## Data Availability

The original contributions presented in the study are included in the article, further inquiries can be directed to the corresponding author.
